# Heating-Enhanced Dielectrophoresis for Aligned Single-Walled Carbon Nanotube Film of Ultrahigh Density

**DOI:** 10.1186/s11671-017-2199-1

**Published:** 2017-06-27

**Authors:** Qingyuan Gu, Maud Guezo, Hervé Folliot, Thomas Batte, Slimane Loualiche, Julie Stervinou

**Affiliations:** 1 The Institute of Physics, Chinese Academy of Sciences, P.O. Box 603, Beijing, 100190 China; 2 FOTON, UMR CNRS 6082, INSA, Avenue des Buttes de Coësmes CS 14315, 35043 Rennes Cedex, France

**Keywords:** Single-walled carbon nanotube (SWCNT), Heating-enhanced dielectrophoresis (HE-DEP), Convection, Alignment density, 88.30.rh, 87.50.ch, 81.15.-z, 06.60.Ei

## Abstract

In this paper, we demonstrate that the alignment density of individualized single-walled carbon nanotubes (SWCNTs) can be greatly improved by heating-enhanced dielectrophoresis (HE-DEP) process. The observations by scanning electron microscope (SEM) suggest ultrahigh alignment density and good alignment quality of SWCNTs. The intuitive alignment density of individualized SWCNTs is much higher than the currently reported best results. The reason of this HE-DEP process is explained by simulation work and ascribed to the heating-enhanced convection process, and the “convection force” induced by the heating effect is assessed in a novel way.

## Background

Single-walled carbon nanotubes (SWCNTs) exhibit strong one-dimensional (1D) polarized properties, as indicates the performance of SWCNT-based devices can be greatly improved by aligning SWCNTs in the same direction. Among the various alignment methods for carbon nanotubes (CNTs), dielectrophoresis (DEP) has been demonstrated to be very efficient and to have the great potential of combining with the large-scale fabrication process of SWCNT-based devices [1, 2]. Numerous studies on DEP were carried out for the alignment or separation of CNTs [1], and high alignment density has been realized [3, 4], but the alignment density is far insufficient for some electronic and photonic applications such as SWCNT-based field-effect transistor (FET) and optical waveguides, in which multilayer aligned SWCNTs, and thus ultrahigh alignment density, are probably required. According to the principle of DEP, the distribution of electric field, the volume of the particles to be aligned, and the complex permittivity of the particles and the solvents with respect to the frequency of the electric field are commonly considered as the major factors in determining the value and direction of DEP force exerted on the particles [5]. Some minor factors, such as the concentration of the particles, the nature of the substrate, and the acting period of the electric field, were also discussed [6–9]. However, all these studies on DEP focus on a static DEP process, without considering the dynamic factors induced by the external effects, such as the convection induced by heating, the fluidity of the solutions and, so on.

Here, we present our work on the heating-enhanced (HE) dynamic DEP process for aligned SWCNT film of ultrahigh density. We think the SWCNTs far from DEP grooves and beyond the DEP force’s ability can be taken to the vicinities of DEP grooves by the convection induced by intentional heating and then are captured by the DEP force, resulting in much higher alignment density of SWCNTs than the case without heating. The simulation work suggests that the heating-induced convection takes the SWCNTs beyond 100 μm far away from DEP grooves to the vicinities of DEP grooves. We assumed that DEP force is equal to the “convection force” at the boundaries of the SWCNTs-gathering areas in the vicinities of DEP grooves, based on which the convection force can be assessed with DEP force.

## Methods

Ten milligrams of pristine HiPCO SWCNT powder was dispersed in the solution of 200 mg sodium cholate (NaCh) in 10 ml deionized water with the help of ultrasonication of 100 W. Then, the mixture was treated by ultracentrifugation of 25 kg for 60 min to remove nanotube bundles. The top layer was extracted and diluted by 100 times as the solution of individually isolated SWCNTs. This SWCNT solution was obtained for the alignment experiment of individualized SWCNT.

**Fig. 1:**
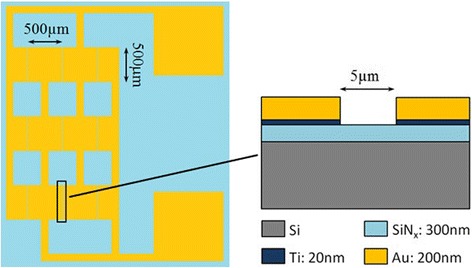
The pattern of DEP chip and the schematic cross section of DEP groove. For the fabrication of the DEP chips, a 300-nm-thick SiN_x_ film was firstly grown directly on silicon substrate by plasma-enhanced chemical vapor deposition (PECVD), and then the SiN_x_ film was covered with a photoresist film made by spin coating method. After baked, the photoresist film was exposed under UV light using the DEP mask and then was developed to remove the exposed photoresist, resulting in the appearance of clear DEP patterns. After the substrate was cleaned and baked again, a 20-nm-thick titanium (Ti) film and a 200-nm-thick gold (Au) film were successively deposited by sputtering. Finally, the unexposed photoresist film together with the Au/Ti film on its surface was removed by acetone, leaving Au/Ti DEP electrodes retained on the exposed area. The width and length of each DEP groove between the electrodes are 5 and 500 μm, respectively. The width of the electrodes is 500 μm

The pattern of one DEP chip and the schematic cross section of one DEP groove with corresponding dimensions are schematically depicted in Fig. 1. For the fabrication of the DEP chips, a 300-nm-thick SiN_x_ film was firstly grown directly on silicon substrate by plasma enhanced chemical vapor deposition (PECVD), and then the SiN_x_ film was covered with a photoresist film made by spin coating method. After baked, the photoresist film was exposed under UV light using the DEP mask and then was developed to remove the exposed photoresist, resulting in the appearance of clear DEP patterns. After the substrate was cleaned and baked again, a 20-nm-thick titanium (Ti) film and a 200-nm-thick gold (Au) film were successively deposited by sputtering. Finally, the unexposed photoresist film together with the Au/Ti film on its surface was removed by acetone, leaving Au/Ti DEP electrodes retained on the exposed area. The width and length of each DEP groove between the electrodes are 5 and 500 μm, respectively. The width of the electrodes is 500 μm.

The DEP experiments were carried out under an AC potential with V_pp_ of 20 V and frequency of 10 MHz for a period of 30 min. Two DEP samples were made. The difference between them is that during the DEP experiment process, one was kept at room temperature (20 °C), and the other was heated at the bottom of the chip by a heating plate with gradual temperature increase from 20 to 100 °C, named as samples A and B, respectively. For each sample, 10 μl SWCNT solution was utilized. Finally, the solution on both samples dried by itself.

## Results and Discussion

**Fig. 2:**
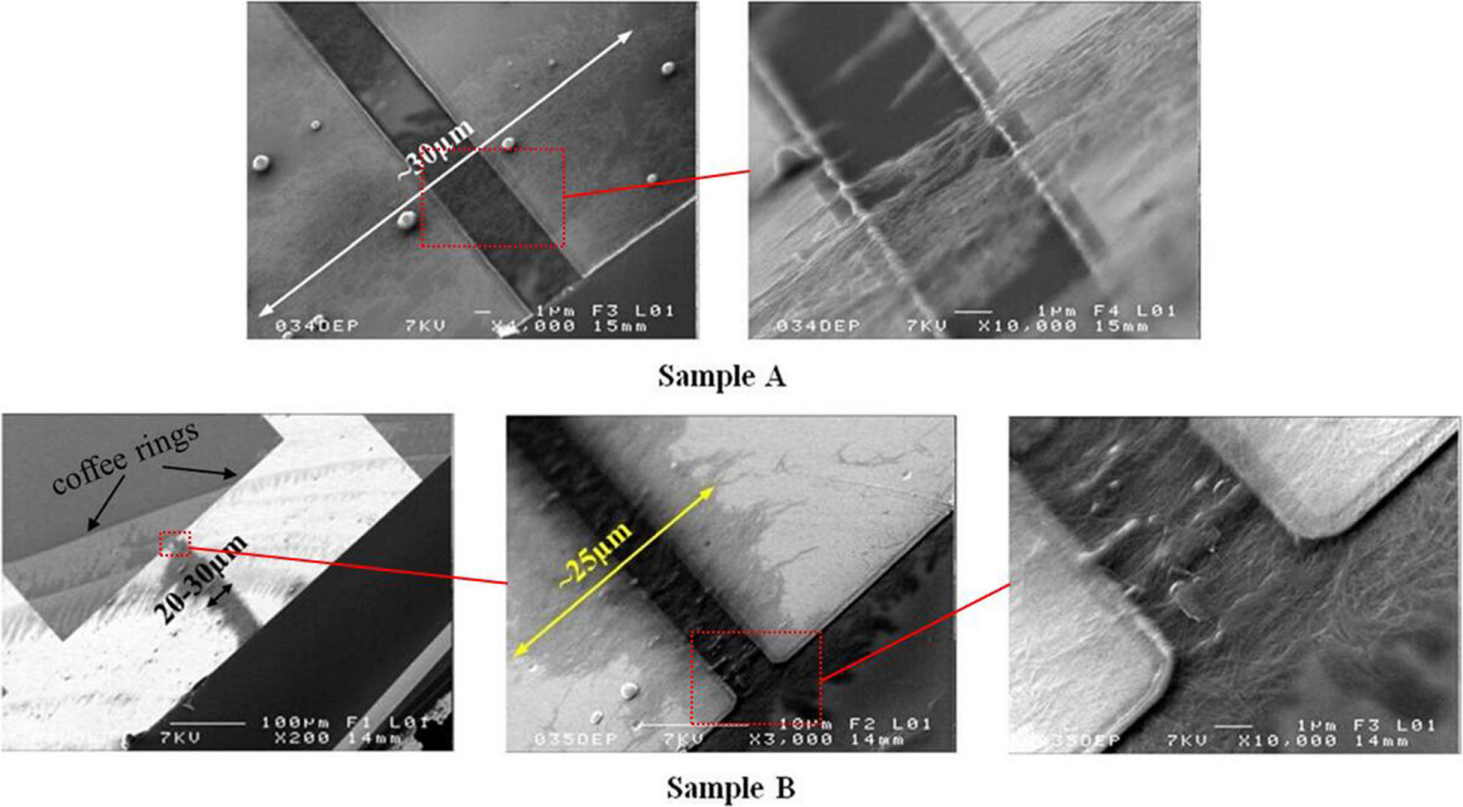
SEM observations of samples A and B. The *red rectangles* indicate the corresponding magnified areas. The *double-headed arrows* present the widths of individualized SWCNT gathering areas. The *two arrows* indicate the coffee rings arising during the drying process of nanotube solution. For sample B, by comparing the contours of coffee rings and SWCNT film in the DEP grooves, we can definitely decide that SWCNT film was formed due to the DEP force induced gathering and alignment but not due to coffee ring effect. The alignment density of individualized SWCNTs on sample B is much higher than that on sample A. The resistances measured betweent the electrodes with aligned SWCNTs are ~20 MΩ for sample A and ~50 KΩ for sample B

The SEM observations of both samples are shown in Fig. 2. The red rectangles indicate the corresponding magnified areas. The double-headed arrows present the widths of individualized SWCNT gathering areas. The two arrows indicate the coffee rings arising during the drying process of nanotube solution. For sample B, by comparing the contours of coffee rings and SWCNT film in the DEP grooves, we can definitely decide that SWCNT film was formed due to the DEP force induced gathering and alignment, but not due to coffee ring effect. By comparison, we can find that the alignment density of individualized SWCNTs on sample B is much higher than that on sample A, so heating enhanced the DEP process on sample B. The intuitive comparison with the highest alignment density currently reported in references [3] and [4] shows that the alignment density of SWCNTs on sample B is also much higher.

The resistances measured between the electrodes with aligned SWCNTs are about 20 MΩ for sample A and about 50 KΩ for sample B. Here, we assume that the widths (5 μm) of DEP grooves are equal to the lengths of the individualized SWCNTs aligned in DEP grooves and that the specific resistances and diameters of all the individualized SWCNTs are the same, and then the resistances between the electrodes are mainly determined by the total cross-sectional area and thus the number of the individualized SWCNTs aligned in DEP grooves with an inversely proportional relation as following:

$$ \frac{R_{\mathrm{A}}}{R_{\mathrm{B}}} = \frac{\kern0.75em \frac{\rho_{\mathrm{SWCNT}}{L}_{\mathrm{SWCNT}}}{S_{\mathrm{A}}}\kern0.75em }{\frac{\rho_{\mathrm{SWCNT}}{L}_{\mathrm{SWCNT}}}{S_{\mathrm{B}}}}=\frac{S_{\mathrm{B}}}{S_{\mathrm{A}}}=\frac{S_{\mathrm{single}\ \mathrm{SWCNT}}{N}_{\mathrm{B}}}{S_{\mathrm{single}\ \mathrm{SWCNT}}{N}_{\mathrm{A}}}=\frac{N_{\mathrm{B}}}{N_{\mathrm{A}}}=\frac{20\ \mathrm{M}\Omega}{50\ \mathrm{K}\Omega}=\frac{400}{1} $$

where *R*, *ρ*, *L*, *S*, and *N* are the resistance, specific resistance, length, cross-sectional area, and number of the individualized SWCNTs in the DEP grooves, respectively. The subscripts A and B denote samples A and B, respectively. We can see the number of the individualized SWCNTs aligned in DEP grooves on sample B is about 400-time higher than that on sample A, so the alignment density of SWCNTs was greatly improved by heating.

In order to analyze the HE-DEP process on SWCNTs, we utilized solid rod-shaped ellipsoid particles to play the roles of the individualized SWCNTs for simulating the DEP force field distribution. In the simulation work, we used the following expression of DEP force ${\overset{\rightharpoonup }{F}}_{\mathrm{DEP}}$ [10, 11]:

$$ \left\langle {\overset{\rightharpoonup }{F}}_{\mathrm{DEP}}\right\rangle =\frac{\pi abc}{3}{\varepsilon}_m\mathrm{R}\mathrm{e}\left(\frac{{\tilde{\varepsilon}}_p-{\tilde{\varepsilon}}_m}{{\tilde{\varepsilon}}_m}\right)\nabla \left[{\left|\mathrm{Re}\left(\nabla \tilde{\phi}\right)\right|}^2+{\left|\mathrm{Im}\left(\nabla \tilde{\phi}\right)\right|}^2\right] $$

where

$$ {\tilde{\varepsilon}}_{p, m}={\varepsilon}_{p, m}-\frac{j{\sigma}_{p, m}}{2\pi \nu}\kern1em \left( j=\sqrt{-1}\right) $$

$$ \tilde{\phi}=\phi \left( x, y, z\right){e}^{i2\pi \nu t} $$

The physical or mathematical significances of all the operators and the parameters, as well as their values used in the simulations for the cases with (100 °C) and without (20 °C) heating, are listed in Table 1, where the values of *ε*_*p*_ and *σ*_*p,m*_ are estimated by referring to reference [12] and considering the conductivity increase of SWCNT solution due to the heating effect and NaCh ionization. For modeling simplification, SWCNTs are regarded as nanorods with length of 1000 nm (*a*) and radius of 1 nm (*b*, *c*), and these value selections are pertinent for our surfactant-wrapped HiPCO SWCNTs.

**Table 1:** The physical or mathematical significances of the operators and the parameters used in the simulations

		SWCNT (*p*)	Water (*m*)
*σ*_*p,m*_	Conductivity of the particles (*p*) and the suspending medium (*m*)	10 S/m	0.2 S/m (20 °C) 0.5 S/m (100 °C)
$\varepsilon$_*p,m*_	Absolute permittivity of the particles and the suspending medium	100 × 8.85 × 10^−12^ F/m	80 × 8.85 × 10^−12^ F/m (20 °C) 55 × 8.85 × 10^−12^ F/m (100 °C)
*a*, *b*, *c*	Half lengths of the major ellipsoid axes	1000 nm, 1 nm, 1 nm	
*v*	Frequency of electric field	10 MHz	
∇	Gradient		
〈〉	Time-average value		
||	Root mean square (RMS)		
$\tilde{\phi}$	Complex electric potential		
Re	Real part of a complex expression		
Im	Imaginary part of a complex expression		
${\tilde{\varepsilon}}_{p,\ m}$	Complex permittivity of the particles (*p*) and the suspending medium (*m*)		

**Fig. 3:**
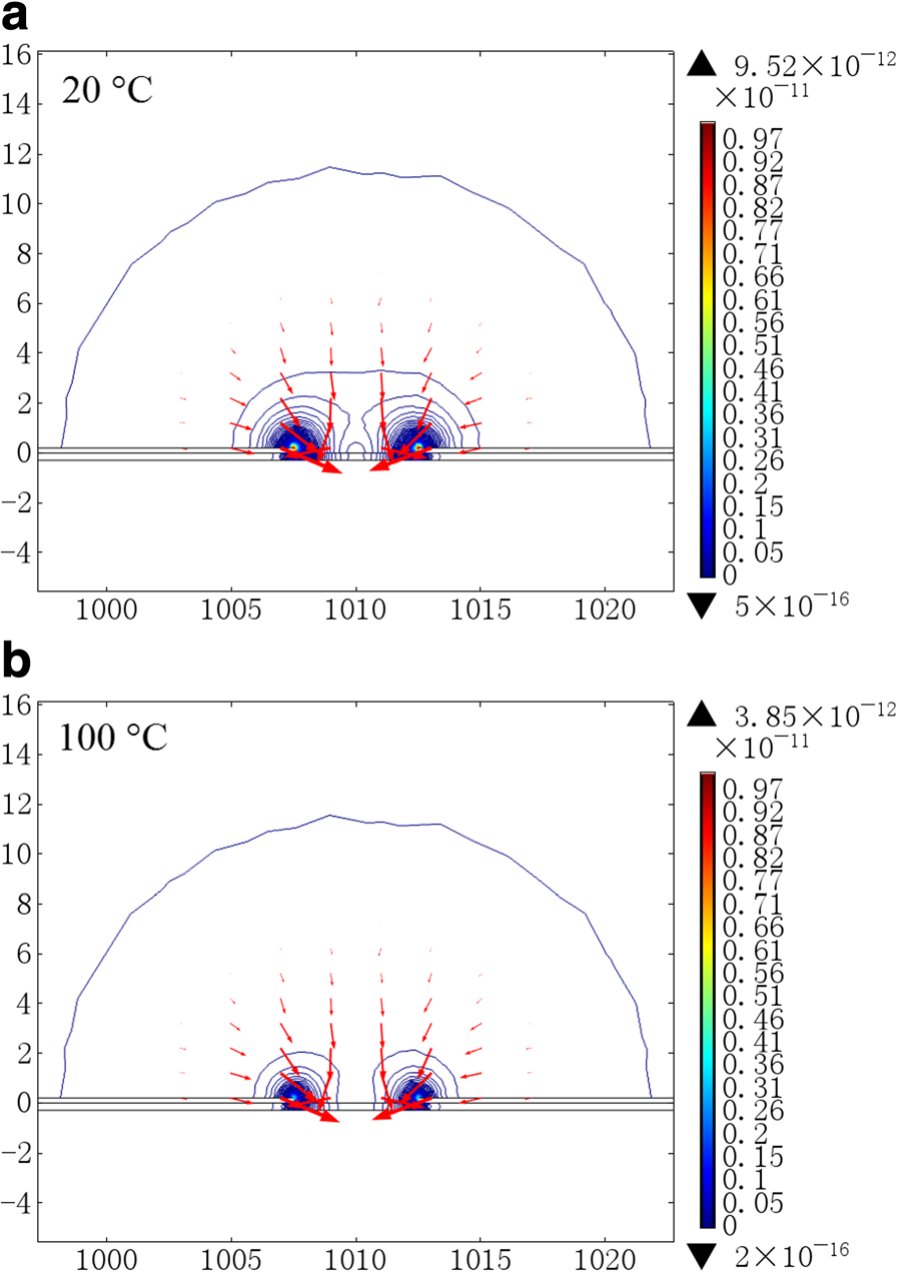
The corresponding DEP force exerted on individualized SWCNTs at 20 and 100 °C, respectively. The *arrows* and the *quasi-hemicycles* denote the direction and value contour of DEP force, respectively. The unit of length is μm. The lengths of the DEP force arrows are proportional to the logarithm of the DEP force value. The outermost quasi-hemicycle contours with a diameter of about 25 μm are corresponding to the DEP force of ~10^−16^ N. The maximum DEP forces are located at the endpoints of the electrodes

The corresponding simulated direction and value contour of DEP force exerted on individualized SWCNTs at 20 and 100 °C are plotted in Fig. 3. The lengths of the DEP force arrows are proportional to the logarithm of the DEP force value. The outermost quasi-hemicycle contours with a diameter of about 25 μm are corresponding to the DEP force of ~10^−16^ N. The maximum DEP forces are located at the endpoints of the electrodes. By comparing the direction and value contour of DEP force in both cases, we can find that the temperature increase from 20 to 100 °C does not lead to significant changes of the order of magnitude of DEP force. It is certain that DEP force only functions in a certain small area and outside this area; DEP force decreases abruptly, as can be reflected by the width of SWCNTs-gathering areas presented by the double-headed arrows in Fig. 2. Outside these areas, SWCNT alignment density is almost zero. Considering the DEP force distribution in Fig. 3, we can find the widths of these areas qualitatively reflect the DEP force values: the bigger the widths are, the bigger the DEP forces are.

**Fig. 4:**
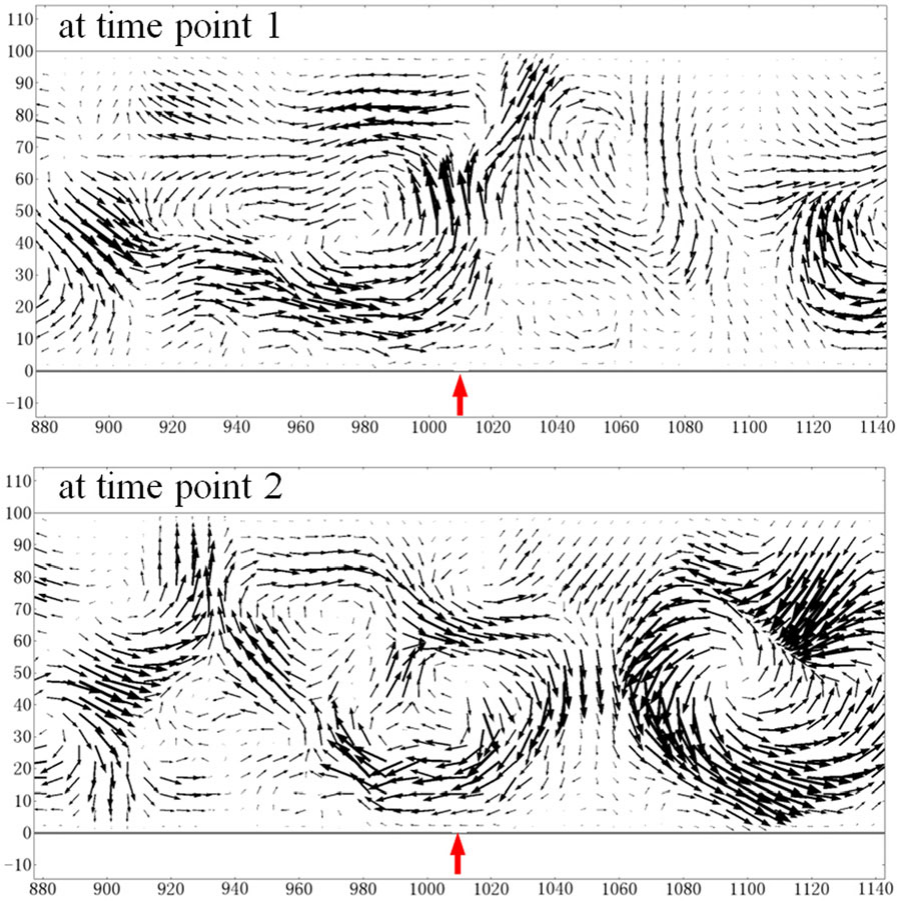
The velocity distribution of natural convection in the SWCNT solution heated at 100 °C. The *red arrows* point out the positions of DEP grooves. At two different time points with an interval of 120 s, the direction of convection is erratic and changes rapidly, and the dimensions of convection vortices are at the level of the depth (100 μm) of SWCNT solution, as indicates that the convection can bring individualized SWCNTs in the dimensions of 100 μm × 100 μm (2D) to the vicinities of DEP grooves

The movement of particles under the heating effect is a rather complex process, in which many forces, including gravitational force, thermophoretic force, viscous drag force, thermodiffusive force, buoyant force, Brownian force and so on, should be considered. To date, there is still no any consensus reached on some of these forces and it is impossible to quantitatively assign their respective specific weight. For simplicity and conciseness, we assign the summation of all these forces excluding DEP force to a convection force so that we can differentiate the DEP process and the convection process during the simulation work. Next, we will demonstrate the DEP process enhanced by the convection force (or convection process) and derive the level of convection force from the DEP force distribution and the deposition or alignment distribution of individualized SWCNTs in the vicinities of DEP grooves.According to the simulation results, thermal equilibration of the SWCNT solution can be quickly reached in 0.2 s when temperature increases from 20 to 100 °C. From the velocity distribution of natural convection in the SWCNT solution heated at 100 °C at two different time points with an interval of 120 s as shown in Fig. 4, we can see that the direction of convection is erratic and changes rapidly, and that the demensions of convection vortices are at the level of the depth (100 μm) of SWCNT solution, as indicates that the convection can bring individualized SWCNTs in the dimensions of 100 μm × 100 μm (2D) to the vicinities of DEP grooves. Moreover, we can also find the exchange and transfer of individualized SWCNTs between neighbor convection vortices, which indicates that individualized SWCNTs farther than 100 μm from DEP grooves can also be brought to the vicinities of DEP grooves. When the individualized SWCNTs far from DEP grooves travel a “long” way with the help of convection and arrive in the vicinities of DEP grooves, where the convection force cannot conquer the strong positive-DEP force (DEP force direction towards the maxima of the electric field), they are captured by DEP force, resulting in the deposition and alignment of these “remote” individualized SWCNTs in the vicinities of DEP grooves, as shown on sample B in Fig. 2. Additionally, the turbulent convection induced by the density difference at different temperatures [http://www.engineeringtoolbox.com/water-thermal-properties-d_162.html] also guaranteed and very high-efficiently enhanced this transfer precess with a velocity in millimeter per second [https://thayer.dartmouth.edu/~d30345d/books/EFM/chap7.pdf]. Contrarily, the absence of both rapidly changing convection vortices and turbulent convection at 20 °C revealed by simulation work suggests the deficiency of SWCNT transfer between different areas, and thus results in the low alignment desity. This is a reasonable explanation for the difference of alignment density between samples A and B in Fig. 2 and thus for the heating-enhanced DEP process. Here, we also strongly claim the repeatibility of this heating-enhanced DEP process.

Based on the above assumptions that the widths of the SWCNTs-gathering areas reflect the DEP force values and that if the convection force cannot overcome the DEP force in the vicinities of DEP grooves, individualized SWCNTs will be seized by the DEP force, we can consider that convection force matches DEP force at the two boundaries of the SWCNTs-gathering areas (20~30 μm as shown in Fig. 2), so it is a new way of assessing the convection force. In the heating (100 °C) case, the simulated DEP force around these two boundaries is in the level of 10^−16^ N (Fig. 3), and thus the convection force should be not far from this value.

We also note that the DEP force at 100 °C is weaker than that at 20 °C (Fig. 3); however, there is almost no change in the widths of SWCNT-gathering areas (Fig. 2). We attibute the reason to the difference of viscous drag force of water at both temperatures (http://www.engineeringtoolbox.com/absolute-dynamic-viscosity-water-d_575.html). Among all the abovementioned convection force elements, viscous drag force plays the most important role in the competition with DEP force, and it presents a decreasing relation with the temperature increase (http://www.engineeringtoolbox.com/absolute-dynamic-viscosity-water-d_575.html). Then consequently, the smaller viscous drag force from water at 100 °C correspondingly requires smaller DEP force, which coincidentally meets the requirement.

Another factor responsible for the increase of individualized SWCNT alignment density may be the gradual increase of SWCNT concentration induced by the evaporation of solvent (water), but according to the algnment results of SWCNTs on sample A, this factor seems not to play a major role. According to the above analysis, the unique significant difference between samples A and B is whether the heating-induced intense convection is present or not, so it is convincing to ascribe the reason of the much higher SWCNT alignment density on sample B to the heating-induced intense convection process, and thus it is forceful to say heating can enhance the DEP process.

## Conclusions

In summary, we have greatly increased the alignment density of individualized SWCNTs by heating-enhanced DEP, in which the heating-induced intense convection plays a crucial role in the exchange and transfer of individualized SWCNTs to the vicinities of DEP grooves where DEP force takes effect to seize SWCNTs. The number of aligned individualized SWCNTs is even enhanced by 400 times. The intuitive comparison shows our alignment density of individualized SWCNTs is much higher than the currently reported best results. This HE-DEP process is explained by the simulation work. We also conceived a new way of assessing the convection force. The realization of ultrahigh alignment density of SWCNTs would be greatly promising for the future performance improvement of SWCNT-film-based devices.
